# Extraction and Characterization of Antioxidants and Cellulose from Green Walnut Husks

**DOI:** 10.3390/foods14030409

**Published:** 2025-01-27

**Authors:** Ivan M. Savić, Ivana M. Savić Gajić

**Affiliations:** Faculty of Technology in Leskovac, University of Niš, Bulevar oslobodjenja 124, 16000 Leskovac, Serbia; savicivana@tf.ni.ac.rs

**Keywords:** waste, ultrasound-assisted extraction, microwave pretreatment, cellulose, Box–Behnken design, UHPLC–ESI–MS/MS method

## Abstract

The ultrasound-assisted extraction process with microwave pretreatment was modeled and optimized to maximize the yield of antioxidants from green walnut husks using a response surface methodology with Box–Behnken design. In this design, the ultrasound-assisted extraction time (10–40 min), ultrasound-assisted extraction temperature (40–60 °C), and microwave pretreatment time (20–60 s) were selected as the factors, while the total antioxidant content was defined as the response. The solvent of choice for extracting antioxidants was 50% (*v*/*v*) ethanol. After optimization using the desirability function, an ultrasound-assisted extraction time of 23 min, ultrasound-assisted extraction temperature of 60 °C, and microwave pretreatment time of 60 s were proposed as the optimal conditions and their validity was verified. Under these conditions, the experimentally determined total antioxidant content was 3.69 g of gallic acid equivalent per 100 g of dry matter. In addition to phenolics, UHPLC–ESI–MS/MS analysis indicated the presence of lipids, quinones, terpenoids, and organic acids in the extract. After the antioxidant extraction, the solid residue was further processed to isolate cellulose in line with the concept of sustainable manufacturing. The structural characterization and hydration properties of cellulose were analyzed to identify its key features and assess its potential for value-added applications. The results demonstrate that green walnut husks are a valuable and cost-effective agro-industrial byproduct for extracting antioxidants and isolating cellulose. This aligns with the principles of a circular economy and the sustainable production of natural compounds.

## 1. Introduction

Walnut is a woody plant that belongs to the Juglandaceae family [1]. It is grown as an industrial plant. Due to its exceptional nutritional properties, the walnut fruit is used in human nutrition [2]. The leaves are used as a medicinal part of the plant and contain various bioactive substances, such as tannins, flavonoids, naphthoquinones (juglone and hydrojuglone), phenolic acid (caffeine and galline), ether oil, vitamin C, and others [3]. This agricultural crop is known to produce large amounts of waste. It is estimated that about 70% of the weight of the fruit is shell and green husk, which represent a cheap raw material. Studies conducted by various authors have demonstrated that green walnut husk extract is a rich source of diverse bioactive compounds, particularly polyphenols [4]. With its unique composition and significant biological activities (antioxidant, antimicrobial, anticancer, etc.) [5], green walnut husk extract is used as a sustainable and environment-friendly dyeing agent [6] and as a substitute for many synthetic compounds (preservatives, antioxidants, flavors) in cosmetic, pharmaceutical, and food products [7]. In addition to their use in these industries, extracts also play a significant role in agriculture, laboratories, and other sectors. The use of green walnut husks can provide large supplies of phytochemicals. In this way, the cultivation of plant material is promoted, which offers multiple environmental benefits, including reducing carbon dioxide in the atmosphere and mitigating the greenhouse effect.

Natural pharmaceutical and cosmetic products are more trusted by consumers because of their safety. For these reasons, there is an increasing need for the use of phytochemicals, especially polyphenols, which have multiple benefits for human health. The available literature describes in detail various operational methods for the extraction of polyphenols from green walnut husk [4]. The selection of the appropriate solvent is a critical factor in preparing extracts that are rich in polyphenols and exhibit strong antioxidant activity. Water, methanol, and ethanol have been recognized as the most suitable solvents for the preparation of green walnut husk extracts [5,8]. In addition to the solvent, the quantity and quality of the extracted polyphenols depend a lot on the extraction technique itself. A wide range of solid–liquid extraction procedures, including conventional [9] and advanced [10,11] techniques, are available for the extraction of polyphenols from walnut husks. However, the conventional extraction procedures have some disadvantages, including low process efficiency, high energy and time consumption, thermal degradation of compounds, negative impact on the environment, and production of extracts of inadequate quality [12]. In order to overcome these shortcomings and improve existing techniques for extracting polyphenols from green walnut husks, advanced extraction methods (ultrasound-assisted extraction, UAE; microwave-assisted extraction, MAE; and supercritical fluid extraction, SFE) were developed [13,14]. These techniques offer tremendous potential to reduce or eliminate the use of toxic organic solvents while increasing extraction yield and extract quality. They are also known as “cold” extraction techniques because the temperature during the extraction process is relatively low and does not affect the stability of the extracted compounds. UAE offers significant cost advantages over SFE due to its lower equipment costs and simpler setup [15]. While SFE requires specialized high-pressure systems and expensive solvents, UAE uses more affordable equipment and can operate under milder conditions, making it a more cost-effective alternative for many extraction applications. Unlike UAE, the main issue with open-vessel microwave-assisted extraction is temperature control, which can lead to the degradation of thermosensitive bioactive compounds [16].

Despite the development of advanced methods for extracting polyphenols from walnut husks, there is still a need for more innovative, efficient, and faster extraction procedures. In this study, the ultrasound-assisted extraction (UAE) combined with microwave pretreatment (MWP) of plant material was modeled and optimized according to the maximal total antioxidant content (TAC). For this purpose, the Box–Behnken design (BBD) was more favorable due to fewer experimental runs and minimal loss of accuracy compared to other optimization designs, resulting in savings of available resources. The three extraction parameters, namely, UAE time, UAE temperature, and MWP time, were optimized. In the optimal extract, the identification of the extracts’ chemical constituents was performed using the chromatographic method, while its antioxidant activity was estimated using the DPPH assay. In line with the principles of the circular economy, which focus on promoting sustainability, reducing environmental impact, and creating new economic opportunities, the second objective of this study was to valorize the solid residue generated after extracting antioxidants from green walnut husks. Cellulose was isolated from the residue due to its biodegradability and renewability, making it a promising alternative to traditional materials.

## 2. Materials and Methods

### 2.1. Chemicals and Reagents

Ethanol (96%, *v*/*v*), sodium carbonate, sodium hydroxide, sodium hypochlorite (Zorka Pharma, Šabac, Serbia), Folin–Ciocalteu’s reagent, gallic acid (97%) (Alfa Aesar, A Johnson Matthey Company, Haysham, UK), acetonitrile, and LC-MS-grade water (Sigma-Aldrich, St. Louis, MO, USA) were used.

### 2.2. Plant Material

Green walnut husks (*Juglans regia* L.) were collected in the territory of southeastern Serbia (Velika Kopašnica, 42°89′ N and 22°02′ E) in October 2019. The plant material was dried at room temperature for 60 days. The moisture content of the plant material was determined gravimetrically by drying at 105 °C to a constant mass. It was found to be 8.1% (m/m), which was in the limited range [17]. The plant material was ground in a laboratory mill (Braun Aromatic KSM2, Kronberg im Taunus, Germany), and a fraction of 0.5 mm was used for antioxidant extraction.

### 2.3. Ultrasound-Assisted Extraction of Antioxidants from Green Walnut Husks with Pre-Treatment

**Figure 1:**
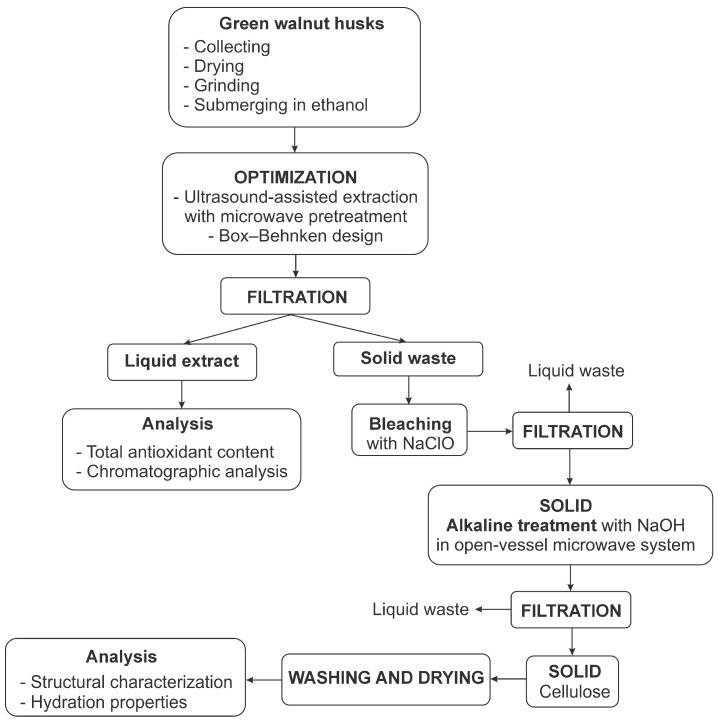
The processes carried out during the extraction of antioxidants from green walnut husks and the isolation of cellulose from solid waste.

In Figure 1, all processes carried out are summarized to provide a better understanding of the whole concept.

The UAE of antioxidants was carried out after the MWP of green walnut husks submerged in 50% (*v*/*v*) ethanol at a liquid-to-solid ratio of 10 mL/g. For MWP treatment, a microwave oven (Vivax MWO-2070 BL) with a constant radiation power of 462 W was used. An ultrasonic bath (Sonic, Niš, Serbia) of 6 L with an operating frequency of 40 kHz and a power of 150 W was a source of ultrasounds. All samples were centrifuged on a TH16B centrifuge (Hong Kong, China) at 6000 rpm for 15 min. The prepared liquid extracts were stored in a refrigerator at 4 °C until further analysis. The UAE was modeled using the BBD with three factors based on the response surface methodology and optimized according to the maximal TAC. TAC represents the extract’s ability to reduce the Folin–Ciocalteu reagent, which is often equated in the literature with the total phenolic content. Górnaś et al. [18] also noted that TAC is preferable because this reagent can be reduced by various bioactive compounds, not just polyphenols. In the BBD, the extraction parameters were analyzed at three factorial levels. The BBD consists of factorial and central points. The central point implies the extraction performed at the mean values of the factor levels in the design space. The analyzed extraction parameters and their coded and actual factor levels are given in Table 1. The UAE time, UAE temperature, and MWP time were included as the extraction parameters, while the TAC was the system response. The samples were pre-treated in a microwave oven to heat the solvent and excite the water present inside the plant cells.

The factors were transferred from the actual value in the coded value using Equation (1):

(1) $$X_{i}=\frac{x_{i}-x_{0}}{\delta_{x_{i}}}$$

where *X_i_* is the coded value of the analyzed factor; *x_i_* and *x*_0_ are the actual and mean values of the analyzed factor, respectively; and *δx_i_* is the step of change for the analyzed factor. A second-order polynomial model (Equation (2)) was analyzed to fit the data and find the best functionality between the response and factors:

(2) $$Y=\beta_{0}+\sum_{i=1}^{n}{\beta_{i}x}_{i}+\sum_{i<j}^{n}\beta_{ij}x_{i}x_{j}+\sum_{j=1}^{n}\beta_{jj}x_{j}^{2}+\varepsilon$$

where *Y* is the response; *x_i_* represents the factors; *β*_0_ is the intercept; *β_i_*, *β_jj_*, and *β_ij_* are the coefficients of linear, quadratic (*x_j_*^2^), and interaction (*x_i_x_j_*) terms, respectively; and *ε* is the residual. The coefficients in the polynomial equations were determined using the least squares method. The model adequacy was confirmed based on the values of the coefficient of determination (R^2^).

### 2.4. Determination of Total Antioxidant Content

The TAC in the ethanolic extracts of green walnut husks was determined spectrophotometrically [19]. The sample was prepared by adding 1 mL of a tenfold diluted Folin–Ciocalteu’s reagent solution in distilled water and 1 mL of 7% (m/V) Na_2_CO_3_ to 0.1 mL of the analyzed extract. After 90 min of incubation, the absorbance of the sample was measured at 765 nm. The TAC is expressed as a gram of gallic acid equivalent per 100 g of dry matter (g GAE/100 g d.m.).

### 2.5. Maceration and Ultrasound-Assisted Extraction Without Pre-Treatment

Maceration (conventional technique) and UAE (advanced technique) were carried out using 50% (*v*/*v*) ethanol at a liquid-to-solid ratio of 10 mL/g to evaluate the effectiveness of UAE–MWP. Maceration was performed at 25 °C in a laboratory flask of 250 mL on a rotary shaker (KS 4000i control, Ika^®^ Werke, Staufen im Breisgau, Germany) at 150 rpm for 24 h. UAE was performed at 60 °C in a thermostated ultrasonic bath (Sonic, Niš, Serbia) with an operating frequency of 40 kHz and a power of 150 W for 30 min. After the extraction, the solid matrix was separated from the liquid extract by vacuum filtration. The content of dry matter was obtained by drying a 3 mL aliquot of the extract in a laboratory oven at 105 °C. The remaining liquid extracts were stored in a refrigerator at +4 °C until further analysis.

### 2.6. Chromatographic Analysis of the Extract

Ultra-high-performance liquid chromatography tandem mass spectrometry with electrospray ionization (UHPLC-ESI-MS) was used for the extract analysis according to conditions given in our previous study with slight modification [20]. The mobile phase was a mixture of water (phase A) and acetonitrile (phase B) acidified with 0.1% (*v*/*v*) formic acid. The following gradient elution was used to separate bioactive compounds: 0–0.8 min (5% B), 0.8–10.8 (5–95% B), 10.8–12.8 min (95% B), 12.8–12.9 min (95–5% B), and 12.9–15 min (5% B). The injection volume of the sample into the system was 5 µL. The mass spectra were recorded in a negative ionization mode with full-range acquisition from m/z 100 to 1000. The identification of bioactive compounds was performed by comparing the masses of quasimolecular and fragment ions with those reported in the literature.

### 2.7. Isolation of Cellulose from Solid Residue After Antioxidant Extraction

Solid residue (50 g) obtained after the antioxidant extraction was treated with 500 mL of 1.4% NaClO adjusted at pH 3 by the acetate buffer. The bleaching process was carried out at 70 °C for 5 h, after which the solid residue was washed with distilled water to achieve a neutral pH. The sample was further treated with 500 mL of 4% NaOH to remove non-cellulose carbohydrates. Alkaline treatment was supported in an open-vessel microwave-assisted extraction system MWO-2070 BL (Vivax, Istanbul, Turkey) at a power of 336 W for 30 min. After cooling, the sample was filtered and washed with distilled water to a neutral pH. At the end of the isolation process, the cellulose was dried and the yield was calculated. The yield of cellulose was expressed in relation to the initial mass of plant material according to Equation (3):

(3) $$Cellulose\;yield\left(\%\right)=\frac{m_{1}}{m_{0}}\times 100$$

where *m*_1_ is the mass of dry isolated cellulose (g) and *m*_0_ is the mass of dry plant material (g).

### 2.8. Structural Characterization of Isolated Cellulose

#### 2.8.1. Fourier Transform Infrared Spectroscopy

Fourier transform infrared spectroscopy (FT-IR) was used for the structural characterization of the isolated cellulose. After the preparation of KBr pellets, the spectrum was recorded in the wavelength range of 4000–400 cm^−1^ with a resolution of 2 cm^−1^ on a Bomem Hartmann and Braun MB-series spectrophotometer (Quebec City, QC, Canada). The obtained spectrum was processed using Win-Bomem Easy software (version 1.9.11, Galactic Industries, Salem, NH, USA). The degree of crystallinity can be determined by comparing the absorption peaks at 1430 cm^−1^ and 900 cm^−1^ [21]. The peak at 1430 cm^−1^ corresponds to the crystalline parts of cellulose, while the peak at 900 cm^−1^ was caused by the amorphous parts. According to Equation (4), the degree of crystallinity (*χ*) was calculated as follows:

(4) $$\chi =\frac{A_{1430}}{A_{900}}$$

#### 2.8.2. Scanning Electron Microscopy (SEM) Analysis

The surface powder morphology of native cellulose was analyzed using a FEI Inspect S50 scanning electron microscope (Hillsboro, OR, USA). A thin layer of sample powder was adhered to an aluminum specimen holder using double-sided tape. Scanning images were captured at magnifications of 239× and 991×, with an accelerating voltage of 10 kV and vacuum of 10^−6^ mbar.

### 2.9. Determination of Hydration Properties of Isolated Cellulose

#### 2.9.1. Determination of Water-Retaining Capacity

The water-retaining capacity (WRC) of cellulose was determined using a previously described [22]. The WRC was calculated using Equation (5):

(5) $$WRC\left(\frac{g}{g}\right)=\frac{m_{2}-m_{1}}{m_{1}}$$

where *m*_1_ is the mass of the dry sample and *m*_2_ is the mass of the sample after soaking in water and centrifuging.

#### 2.9.2. Determination of Water Swelling Capacity of Cellulose

The water swelling capacity (WSC) of isolated cellulose was determined by soaking 0.5 g of dry cellulose in 20 mL of distilled water at room temperature [23]. After 24 h, the volume of swollen cellulose was measured. The WSC was calculated using Equation (6):

(6) $$WSC\left(\frac{mL}{g}\right)=\frac{V_{1}-V_{0}}{m_{1}}$$

where *V*_1_ is the volume of swollen cellulose (mL); *V*_0_ is the volume of dry cellulose (mL); and *m*_1_ is the mass of cellulose (g).

### 2.10. Statistical Analysis

All data are presented as mean ± standard deviation of three measurements and processed statistically in IBM SPSS Statistics 27 software (International Business Machines Corporation, Armonk, NY, USA). Design Expert 13.0.1.0 (Stat-Ease, Minneapolis, MN, USA) was used to generate the design matrix and regression models. An ANOVA test was performed for the proposed polynomial model at the confidence level of 95%. The statistical significance of the terms in the polynomial model was estimated based on the F-value.

## 3. Results and Discussion

### 3.1. Modeling the Ultrasound-Assisted Extraction of Antioxidants from Green Walnut Husks

UAE is the most commonly used advanced extraction technique. It is very efficient and requires a shorter extraction time than traditional techniques. In this study, the possibility of applying microwave pretreatment to reduce the time of exposure to ultrasound effects and enable obtaining a high-quality extract was considered. Microwaves rapidly heat the solvent chosen for the extraction, resulting in better solubility of the desired compounds during UAE. The UAE time was observed in the range of 10–40 min, while the UAE temperature was between 40 °C and 60 °C because higher temperatures cause a decrease in TAC [8]. An MWP time of up to 60 s was taken into account because heating the system for a longer time may cause the degradation of the desired compounds. A total of 17 extractions were performed following the BBD matrix, whose order is shown randomly in Table 2. The TAC in the extracts varied in the range of 2.49–3.70 g GAE/100 g d.m. The lowest TAC was observed in the extract obtained after 10 min of ultrasonic treatment and at an extraction temperature of 40 °C with 40 s of MWP of the plant material. The extract obtained after 25 min of ultrasonic treatment at 60 °C and 60 s of MWP of the plant material had the highest TAC of 3.70 g GAE/100 g d.m.

The obtained TAC was modeled by a second-order polynomial model, which can be represented in terms of coded factors using Equation (7):

(7) $$Y=3.320+0.064X_{1}+0.307X_{2}+0.265X_{3}-0.105X_{1}X_{2}-0.048X_{1}X_{3}-0.145X_{2}X_{3}-0.312X_{1}^{2}-0.065X_{2}^{2}+0.008X_{3}^{2}$$

where *Y* is the TAC; *X*_1_ is the UAE time, *X*_2_ is the UAE temperature; and *X*_3_ is the MWP time. The positive coefficients in the polynomial equation led to an increase in TAC with increasing factor levels and vice versa. A higher value of the regression coefficient indicated a greater influence of the extraction parameter on the defined response. The analysis of linear terms suggested that all extraction parameters positively influence the TAC. UAE temperature had the greatest impact of all analyzed factors, followed by MWP time and UAE time. The influence of UAE time was many times smaller than that of the other two extraction parameters.

In Table 3, the results of the ANOVA test for the second-order model are presented. The model’s F-value of 79.30 was highly significant, with only a 0.01% probability of it being due to random variation. The interaction between UAE time and MWP time was statistically insignificant (*p* > 0.05) and can be excluded from the equation to improve the model’s prediction ability. The lack-of-fit F-value of 0.27 was below the critical F-value of 6.59, indicating it is insignificant when compared to the pure error (0.0157). Otherwise, the lack of fit corresponds to the model’s error, while the pure error indicates the experimental error. The statistically insignificant lack of fit is a confirmation of model adequacy [24].

**Figure 2:**
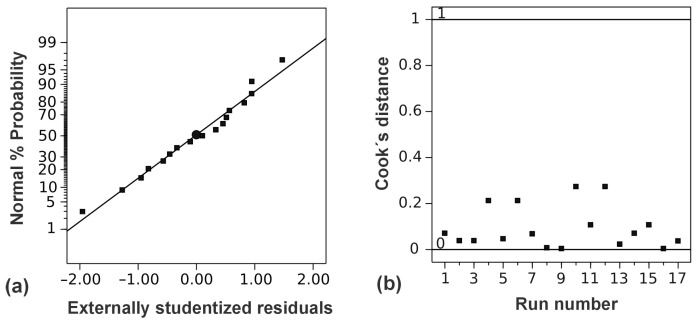
Diagram of normal distribution of studentized residuals (**a**) and Cook’s distance (**b**) for the second-order polynomial model.

A coefficient of variation of 1.65% is favorable, as lower values are preferred [25]. Additionally, the adequate precision, which measures the signal-to-noise ratio, was 30.71. The R^2^ of 0.990 implied that 99% of the variation in the TAC can be explained by the proposed regression model. Given that the difference between the predicted R^2^ (0.961) and the adjusted R^2^ (0.978) was less than 0.2, and the adequate precision was far greater than 4 [26], the proposed model can be used for navigation in the design space. In Figure 2a, the normal probability plot of studentized residuals is depicted. The data exhibited linear dependence, resulting in normally distributed residuals. With Cook’s distances all below 1, there were no significant points or outliers (Figure 2b).

**Figure 3:**
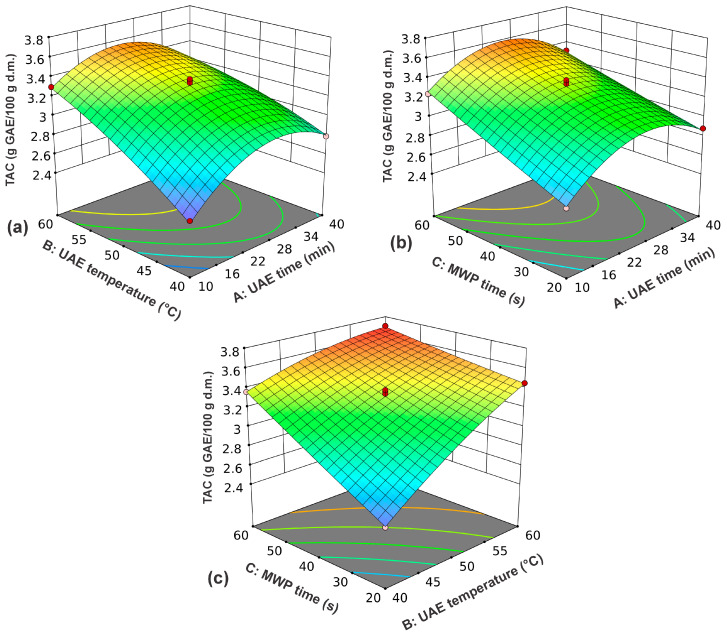
The influence of UAE time and UAE temperature for an MWP time of 40 s (**a**); UAE time and MWP time at a UAE temperature of 50 °C (**b**); and UAE temperature and MWP time for a UAE time of 25 min (**c**) on the TAC of green walnut husks.

In Figure 3, the influence of UAE time and UAE temperature on TAC is depicted in the form of a three-dimensional plot. As UAE time increased, TAC initially increased, reaching its peak at 25 min [27], after which it began to decline (Figure 3a). This behavior is likely due to the degradation of bioactive compounds caused by prolonged exposure to cavitation energy. As the UAE temperature increases, the TAC also rises [27], with this effect being significantly more pronounced at shorter UAE times. Soto-Maldonado et al. [8] monitored the effect of temperature on the TAC of walnut shell extract and observed the highest value at 50 °C. This research has shown that the solubility of antioxidants increases significantly at moderate temperatures without their thermodegradation. A strong interaction between UAE time and UAE temperature was not observed. The interaction between UAE time and MWP time at 50 °C is given in Figure 3b. The plot indicates that extending the MWP time results in a higher yield of antioxidants. The yield of antioxidants was significantly higher for longer MWP times. This type of pretreatment of the plant material facilitated the heating of the solvent and the agitation of intracellular water, resulting in the rupture of cell walls and the release of intracellular contents into the solvent [13]. In Figure 3c, the influence of UAE temperature and MWP time on TAC for a UAE time of 25 min is given. The impact of UAE temperature was more noticeable during shorter UAE durations, but it diminished significantly over longer periods. The TAC increased with changes in MWP time, but this effect was more pronounced at lower UAE temperatures.

### 3.2. Optimization of Antioxidant Extraction from Green Walnut Husks

The UAE–MWP of the plant material was optimized according to the maximal TAC in green walnut husk extract using a desirability function. With this approach, the following optimal conditions were obtained: UAE time of 23 min, UAE temperature of 60 °C, and MWP time of 60 s. The extraction was carried out under these proposed conditions to check their validity. The experimental TAC of 3.64 g GAE/100 g d.m. and predicted TAC of 3.69 g GAE/100 g d.m. were in good agreement and indicated data validity. Soto-Maldonado et al. [8] also described the extraction of antioxidants from green walnut husks according to a traditional procedure. They monitored the influence of solvent polarity (ethanol, methanol, and acetone) and temperature on TAC at a liquid-to-solid ratio of 20 mL/g. The optimal temperature for the extraction of antioxidants was determined to be 50 °C, as it enhanced the solubility of the compounds without causing thermal inactivation or degradation. With respect to solvent polarity, increasing the water content in the pure solvent resulted in a higher TAC. In the case of 50% (*v*/*v*) ethanol, the TAC was less than 15 mg GAE/g d.m. even after 4 h of maceration extraction. By comparing the TAC obtained in our study with their results, it can be concluded that the proposed procedure achieves a significantly higher yield in a shorter extraction time. Soto-Maldonado et al. [8] analyzed the effect of liquid-to-solid ratio and ethanol concentration on the UAE (600 W, 20 kHz, 25 °C, 40 min) of antioxidants from walnut green husks. The highest TAC of 100 mg GAE/g d.m. was achieved at a liquid-to-solid ratio of 25 mL/g and for 75% (*v*/*v*) ethanol, which was about 3.5 times lower than in our study. Also, taking into account the liquid-to-solid ratio and the extraction time, our procedure can be considered very effective for the recovery of antioxidants from green walnut husks.

### 3.3. Comparison of Extraction Techniques

The maceration of antioxidants was conducted to compare the traditional technique with the proposed procedure, while UAE was performed to examine the impact of MWP treatment of plant material on TAC (Table 4). The lowest TAC of 1.96 g GAE/100 g d.m. was determined in the extract obtained by maceration, while a higher TAC was obtained using UAE. The UAE–MWP method achieved a TAC that was about 50% higher than maceration and 10% higher than UAE, all in a shorter time. The higher TAC is attributed to the synergistic effect of microwave and ultrasound radiation in the proposed method, which enhances the destruction of plant cells and the release of antioxidants. The total electricity consumption of 0.066 kWh for the proposed method at the laboratory level was lower compared to the consumption during UAE. Considering the shorter extraction time and lower electricity consumption, the proposed method for the extraction of antioxidants from green walnut husks can be deemed energy-efficient at the laboratory level.

Barekat et al. [4] prepared the extracts of green walnut husks of various cultivars from Iran using UAE with ice water for 60 min and determined the TAC in the range of 35–58 mg/g GAE/g d.m. Momen and Satari [28] also determined the TAC in the extracts of green walnut husks from Iran using microwave-assisted extraction (power of 850 W and extraction time of 5 min) and UAE (frequency of 28 kHz, power of 100 W, and extraction time of 22 min). In both cases, the TAC was 220 mg/g GAE/g d.m., which is significantly higher compared to the content obtained in the previous study. Xi et al. [29] reported that the TAC in ethyl acetate polar fraction of husk was about 48 mg GAE/g d.m. By analyzing and comparing the obtained data with those available in the literature, it can be concluded that, in addition to the plant’s type and growing conditions, the extraction technique and operating conditions significantly impact the antioxidant yield.

### 3.4. UHPLC–MS/MS Analysis of the Extract

**Figure 4:**
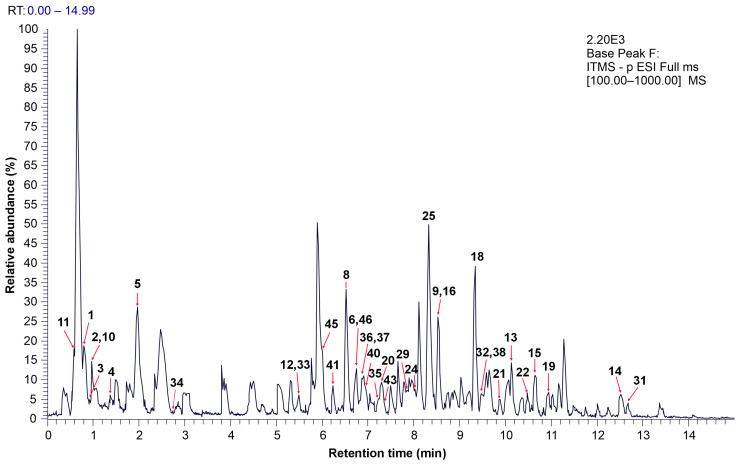
The base peak chromatogram of green walnut husk extract obtained under optimal conditions for antioxidant extraction. The peak number corresponds to the detected compound, as listed in Table 5. NL—normalization level; ITMS—c; ESIA—ion trap mass spectrometry combined with electrospray ionization.

The optimal extract was subjected to chromatographic analysis to identify the bioactive compounds. The chromatogram according to the base peak in the mass range of 100–1000 is depicted in Figure 4. A total of 49 compounds were detected, of which 13 were not identified (Table 5). Phenolic compounds were the most prevalent among the identified compounds, followed by lipids. The presence of quinones, terpenoids, and organic acids was also confirmed.

#### 3.4.1. Phenolic Compounds

Polyphenols are the most diverse and widespread secondary plant metabolites; they occur in free form or more commonly as glycosides. These compounds boast a remarkable range of pharmacological activities and typically contribute positively to overall health [51]. In contrast, the growing consumer focus on environmental protection, sustainable development, and the desire to maintain youth and health have caused an increasing interest in phenolic compounds. These compounds are used in the production of functional foods [52], dietary supplements [53], and cosmetic products [54]. When selecting such products, consumers expect greater safety for human health and the environment, high quality, and efficiency that matches or exceeds that of synthetic compounds.

The optimal extract contained various phenolic compounds, including phenolic aldehydes, phenolic acids, stilbenoids, tannins, and flavonoids. Compound 2 with [M–H]^−^ ion at m/z 136.55 was identified as a protocatechuic aldehyde (phenolic aldehyde). All identified phenolic acids (compounds 3, 5, 8, 11, 33, 37, and 38) belong to hydroxycinnamic acids. The quasimolecular adduct ion, [M–H+HCOOH]^−^, at m/z 225.04 (t_R_ 0.59 min, compound 11) originated from caffeic acid, and the peak with [M–H]^−^ ion at m/z 180.91 corresponded to its dihydro form (compound 5). A caffeic acid derivative (compound 33) corresponded to the peak with [M–H]^−^ ion at m/z 431.13 and t_R_ 5.47 min. Based on [M–H]^−^ ion at m/z 208.98 and fragment ions at m/z 165 (100%) and 137, compound 8 was identified as hydroxy ferulic acid. The peak at t_R_ 6.82 min with [M+HCOOH]^−^ at m/z 517.12 belonged to *p*-coumaric acid glycoside (compound 37), and the peak at t_R_ 9.52 min with [M–H]^−^ ion at m/z 535.12 originated from *p*-coumaroyl derivative (compound 38). The peaks originating from *cis*-resveratrol (compound 12, stilbenoid) and valenoic acid dilactone (compound 35, hydrolyzable tannin) corresponded to [M–H]^−^ ions at m/z 227.04 and m/z 469.19, respectively. The extract also contained 13 different flavonoids, including flavanonols, flavonols, and flavones.

Compound 14 with [M–H]^−^ ion at m/z 271.19 and compound 16 with [M–H]^−^ ion at m/z 287.18 were identified as two different flavanonols, namely pinobanksin and fustin, respectively. Among the identified flavonols, only compound 20 was present in its pure form. This compound was identified as quercetin, based on the [M–H]^−^ ion at m/z 301.06 and its fragment ions at m/z 273, 179, 151, and 107. The presence of this compound was also confirmed in 50% (*v*/*v*) methanolic extract of green walnut husks prepared by the UAE at 50 °C for 45 min [45]. Compound 22, identified as an isorhamnetin derivative, had [M–H]^−^ ion at m/z 315.29. The peaks that had [M–H]^−^ ions at m/z 401.05, m/z 429.12, and m/z 577.12 originated from three different apigenin derivatives (compounds 29, 32, and 40). Sheng et al. [45] and Medic et al. [55] reported that green walnut husk extract contains kaempferol glucoside. Compounds 41 ([M–H]^−^ ion at m/z 593.22), 45 ([M–H]^−^ ion at m/z 739.27), and 46 ([M–H]^−^ ion at m/z 767.29) were identified as kaempferol derivatives. Acacetin-rhamnoglucoside isomer (compound 43) was the only identified flavone with [M–H+HCOOH]^−^ ion at m/z 637.03. Based on the analysis, green walnut husk extract can be considered a valuable source of various phenolic compounds, which were also confirmed in many other studies [45,55].

#### 3.4.2. Lipids

Among lipids, only fatty acids were identified. According to their content, fatty acids were in second place after phenolic compounds. The peak at t_R_ 6.76 min with a [M–H]^−^ ion at m/z 187.05, indicated the presence of azelaic acid, a medium-chain fatty acid (compound 6). Compound 13, oxidized fatty acid, was identified at t_R_ 10.12 min with [M–H]^−^ ion at m/z 265.10. Compounds 15, 18, and 19 were identified as polyunsaturated fatty acids, whereas compounds 21, 24, and 25 belonged to monounsaturated fatty acids. Among these unsaturated fatty acids, only compound 15 ([M–H]^−^ ion at m/z 279.16) was in pure form. Other fatty acids (compounds 21, 24, and 25) were identified as hydroxy forms. Unsaturated fatty acids are extremely important in human nutrition because they have a positive effect on their health. They enhance the properties of skin and hair, slow the aging process, boost immunity, regulate blood sugar levels, and offer protection against cardiovascular diseases, cancer, stroke, etc., [56]. For these reasons, the extract can be considered highly nutritious. Romano et al. [14] also demonstrated that green walnut husk extract prepared by supercritical extraction with carbon dioxide is rich in unsaturated fatty acids, particularly linoleic acid.

#### 3.4.3. Quinone

Juglanosides and their derivatives are common constituents of *Juglans regia* species and have been identified in other parts of this plant in addition to the husk [45]. One of the identified compounds was the juglanoside D derivative (compound 31), which exhibited [M–H]^−^ ion at m/z 427.21.

#### 3.4.4. Terpenoids

The quasimolecular [M–H]^−^ ion at m/z 457.19 (compound 34) and its adduct [M–H+HCOOH]^−^ ion at m/z 503.05 (compound 36) originated from a dihydrophaseic acid derivative (terpenoid). Wang et al. [57] concluded that the most abundant compounds were terpenoids in 50% (*v*/*v*) ethanolic extract of green walnut husks. However, a thorough review of the literature revealed that the composition of terpenoids varies in different extracts. This variation can be attributed to the plant’s cultivation location and the extraction conditions [14].

#### 3.4.5. Organic Acids

Four different organic acids (compounds 1, 4, and 10) were identified in the extract. The [M–H]^−^ ion at m/z 132.94 corresponded to malic acid (compound 1). Vieira et al. [30] confirmed its presence in 80% (*v*/*v*) ethanolic extract of green walnut husks, obtained by stirring at room temperature (25 °C) for 60 min. (–)-Citramalic acid (compound 3) was confirmed based on [M–H]^−^ ion at m/z 146.94 and its main fragment ion at m/z 87. Compound 4 ([M–H]^−^ ion at m/z 160.92) was identified as 1,1,2-ethanetricarboxylic acid, while [M–H+HCOOH+HOH]^−^ ion at m/z 224.93 was the result of 3- or 4-hydroxy-2-oxoglutaric acid (compound 10).

### 3.5. Cellulose Isolation from Solid Residue After Antioxidant Extraction

The conventional procedure for the isolation of cellulose from plant material consists of several stages. The first stage is the defatting of the plant material to remove fat and fat-soluble substances. The second stage is bleaching to remove lignins, while the third stage is alkaline treatment, which can remove and hydrolyze hemicellulose, silica, and ash [23]. Sodium hydroxide is commonly used for alkaline treatment of plant material. Han and Geng [58] observed that sodium hydroxide concentration and temperature significantly affect cellulose content, purity, and yield. At lower concentrations of sodium hydroxide, monomolecular water is formed, enabling the penetration of NaOH dipole. This process can be enhanced by increasing the alkali concentration [23]. These conditions also contribute to the destruction of the amorphic surface of cellulose. Temperature affects the creation of high-energy ions that can strongly penetrate deeper layers of cell walls, resulting in an easier removal of pectins, lignins, and hemicellulose. Careful control of alkali concentration and pH value is crucial for alkaline treatment. Based on the literature data, a 4% NaOH solution was identified as the optimal agent. The alkaline treatment takes a long time (from a few hours up to 24 h) and is energetically demanding [59]. In this study, the process was assisted by microwaves to speed up and make it more efficient. The total extraction time of the alkaline treatment was 30 min. Following the antioxidant extraction, the cellulose yield was calculated and found to be 28.3 ± 0.34%. The cellulose content of this agricultural by-product has not been described in detail in the literature. However, about 35% is known to be available in the shell [60]. Although the yield of isolated cellulose was lower compared to some other agro-industrial wastes [61], green walnut shells can be considered a valuable source of cellulose. The whole approach is according to the concept of the application of pure and green technologies that are ecologically acceptable and sustainable [62]. The isolated cellulose was a white, odorless, and tasteless powder with a density of 1.3 g/mL.

### 3.6. FT-IR Analysis of Isolated Cellulose

**Figure 5:**
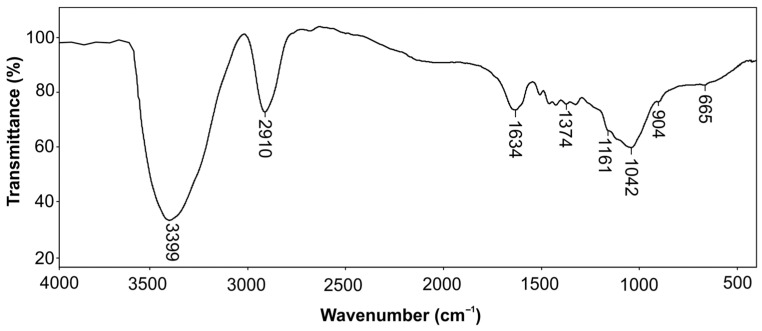
Infrared spectrum of isolated cellulose from solid residue after the extraction of antioxidants from green walnut husks.

In Figure 5, the FT-IR spectrum of isolated cellulose from solid residue after the extraction of antioxidants from green walnut husks is presented. The band at 3339 cm^−1^ originated from the valence vibration of the –OH group, while the bands at 2910 cm^−1^ and 1374 cm^−1^ were the result of the valence vibration of the C–H bond of glucose. The vibrations of water absorbed in cellulose gave the a at 1634 cm^−1^ [23], while the valence vibration of the C–O–C bond produced a band at 1161 cm^−1^. The deformation vibration of the –CH_2_ group and C–H bond was noted at 1431 cm^−1^ and 1375 cm^−1^, respectively. The band at 904 cm^−1^ was characteristic of the *β*-glycosidic bond between the glucose unit. This band, which corresponds to the amorphous region, is often used to identify cellulose in various biological and synthetic materials. The spectrum was in accordance with the cellulose commercial whose spectrum has described in the literature [63]. The spectrum of isolated cellulose was similar to that of commercial cellulose, with no new bands observed, but with slight variations in their intensity and position. This indicates that there were no changes in the functional groups, suggesting that they were not chemically modified during the isolation process. The band originated from the valence vibration of the O–H group and was shifted to the lower wavelengths (3399 cm^−1^) compared to the commercial cellulose (3509 cm^−1^). It was probably due to the formation of intramolecular or intermolecular hydrogen bonds, conformation change, and interchain interactions in cellulose. The similar intensity of the bands at 898 cm^−1^ and 904 cm^−1^ in the spectra of commercial and isolated celluloses suggests the presence of the same amorphous region.

The ratio of band intensity at two specific wavelengths, around 1430 nm and 900 nm, was used to calculate the degree of crystallinity [64]. If this value is approximately 1, the degree of crystallinity of the analyzed sample can be considered high. In our case, the degree of crystallinity of 0.93 indicated a high crystalline structure, high purity, and material stability. The reduced band splitting of the bands in the C–H vibration region of methyl and methylene groups (2850–2910 cm^−1^) also suggested the successful removal of lignin during cellulose isolation. The spectroscopic analysis showed that the operative conditions (high temperature and alkali concentration) enabled the removal of non-cellulose materials, including hemicellulose and lignin. The satisfactory purity and quality of the isolated cellulose were consistent with the results of He et al. [23].

### 3.7. SEM Analysis of Isolated Cellulose

**Figure 6:**
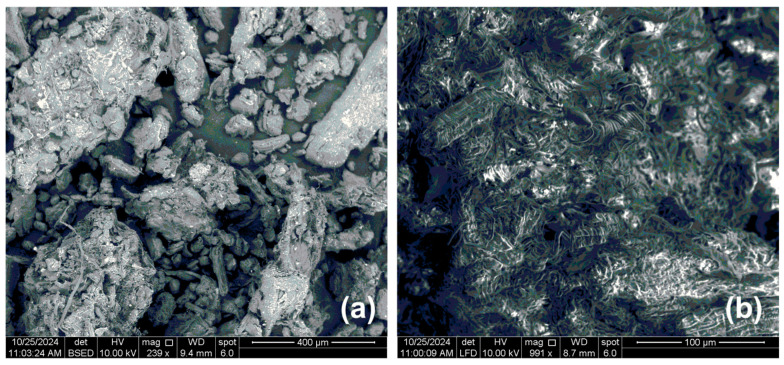
Scanning electron microscope images of isolated cellulose from green walnut husks at magnifications 239× (**a**) and 991× (**b**).

In Figure 6, the SEM images of native cellulose powder obtained by the different magnifications are presented. The cellulose surface at the magnification 239× is almost flat with spherical and cylindrical particles (Figure 6a). The porous structure and agglomerate of fibrous structure of cellulose powder can be noticed in the SEM image with magnification 991× presented in Figure 6b. Bhandari et al. [65] and Prasanna and Mitra [66] also confirmed using SEM analysis that native cellulose typically exhibits a fibrillar or fibrous morphology, depending on the source and isolation method. Uranchimeg et al. [67] showed that the morphology of cellulose significantly impacts the grinding process. The porous structure of isolated cellulose makes it highly effective for various filtration and separation processes, as well as for biomedical applications and composite material fabrication.

### 3.8. Hydration Properties of Isolated Cellulose

WRC and WSC are important parameters for the evaluation of cellulose quality since the hydration properties are related to its structure. Cellulose consists of a lot of hydrophilic groups that contribute to its high WRC and WSC [66]. Therefore, it is recommended for human nutrition as it helps reduce pressure in the intestinal tract and eliminate toxins [68]. The determination of WRC is important for evaluating the reactivity of cellulose, i.e., the availability of its hydroxyl groups. The WRC of isolated cellulose was 4.01 ± 0.043 g/g, indicating a satisfactory ability to retain water due to the presence of a greater number of hydrophilic groups. He et al. [69] and Khan et al. [70] also reported that isolated cellulose from agro-industrial waste had a good WRC, although this parameter may vary depending on the source of the plant material and the extraction technique.

Although cellulose has a lower WSC compared to other polymers, it still exhibits a degree of swelling under certain conditions, which is important for various industrial and biomedical applications. The determined WSC was 8.4 ± 0.15 mL/g, while cellulose swelled to about 38% of its initial volume at room temperature. This was consistent with the swelling capacity of between 20% and 40% described by Ottesen and Syverud [71]. Interestingly, the WSC of our cellulose was similar when compared to cellulose from cocoa pod husk [72].

Based on the obtained data, it can be concluded that isolated cellulose can be used as the initial material for various industrial applications. One of the key applications of isolated cellulose can be as a stabilizer or thickener in food products [73]. Its water-retaining properties can enhance texture and extend product shelf life while lowering fat and calorie content. Additionally, isolated cellulose may serve as a potential pharmaceutical excipient in the industry [72].

## 4. Conclusions

The proposed UAE–MWP of green walnut husks was presented as an effective and innovative method for antioxidant extraction. This approach offered significant advantages over traditional maceration and UAE without MWP, including a reduced extraction time and higher yields, making it a viable option for industrial-scale applications. The ethanolic extract of green walnut husks enriched with antioxidants (TAC of 3.64 g GAE/100 g d.m.) contained phenolics, quinones, terpenoids, organic acids, and lipids identified using the UHPLC-ESI-MS/MS method. It can contribute to improving the nutritional composition and shelf-life of products. Additionally, the solid residue after the extraction of antioxidants can be further used for cellulose isolation. The determined hydration properties (WRC and WSC) indicated that cellulose can be a useful raw material for manufacturing various products. In short, this approach not only promotes the use of renewable resources but also encourages the cultivation of this plant species, contributing to reducing the greenhouse effect.

## Figures and Tables

**Table 1:** Coded (−1, 0, +1) and uncoded values of factor levels.

Factor	Factor Levels		
	−1	0	+1
UAE time (min), *X*_1_	10	25	40
UAE temperature (°C), *X*_2_	40	50	60
MWP time (s), *X*_3_	20	40	60

**Table 2:** Box–Behnken design matrix with experimental and predicted values of total antioxidant content.

Std.	Run	A: UAE Time (min)	B: UAE Temperature (°C)	C: MWP Time (s)	TAC (g GAE/100 g d.m.)	
					Experimental	Predicted
3	1	10	60	40	3.42	3.29
12	2	25	60	60	3.70	3.69
9	3	25	40	20	2.53	2.54
7	4	10	50	60	3.24	3.26
16	5 *	25	50	40	3.68	3.32
6	6	40	50	20	2.88	2.86
13	7 *	25	50	40	2.84	3.32
15	8 *	25	50	40	3.34	3.32
11	9	25	40	60	2.92	3.36
1	10	10	40	40	2.49	2.46
5	11	10	50	20	2.52	2.64
4	12	40	60	40	3.18	3.21
14	13 *	25	50	40	3.36	3.32
2	14	40	40	40	2.79	2.80
8	15	40	50	60	3.31	3.29
10	16	25	60	20	4.27	3.45
17	17 *	25	50	40	3.16	3.32

Std.—standard order; *—central point; TAC—total antioxidant content.

**Table 3:** ANOVA test for the second-order polynomial model.

	Sum of Squares	df	Mean Square	F-Value	*p*-Value
Model	1.9256	9	0.2140	79.30	3.28·10^−6^ *
A—UAE time	0.0331	1	0.0331	12.27	9.96·10^−3^ *
B—UAE temperature	0.7550	1	0.7550	279.84	6.67·10^−7^ *
C—MWP time	0.5624	1	0.5624	208.45	1.82·10^−6^ *
AB	0.0437	1	0.0437	16.21	5.02·10^−3^ *
AC	0.0092	1	0.0092	3.40	0.11 **
BC	0.0841	1	0.0841	31.16	8.31·10^−4^ *
A^2^	0.4096	1	0.4096	151.82	5.32·10^−6^ *
B^2^	0.0176	1	0.0176	6.54	0.04 *
C^2^	0.0003	1	0.0003	0.10	0.76 **
Residual	0.0189	7	0.0027		
Lack of fit	0.0032	3	0.0011	0.27	0.85 **
Pure error	0.0157	4	0.0039		
Total correction	1.9445	16			
Std. Dev.	0.052		R^2^		0.990
Mean value	3.14		adjusted R^2^		0.978
C.V.%	1.65		predicted R^2^		0.961
			Adequate precision		30.71

*—statistically significant; ** –statistically insignificant; df—degree of freedom; Std. Dev.—standard deviation; C.V.—coefficient of variation; R^2^—coefficient of determination.

**Table 4:** Total antioxidant content of green walnut husk extract obtained by different extraction techniques.

Extraction Technique	Extraction Temperature [°C]	Extraction Time	TAC [g GAE/100 g d.m.]	Energy Consumption [kWh]
Maceration	25	24 h	1.96	0
UAE	60	30 min	3.32	0.075
MWP–UAE	unknown/60 °C	1 min/23 min	3.64	0.008/0.058

**Table 5:** Identification of bioactive compounds in the optimal extract of green walnut husks.

No.	Quasimolecular or Adduct ion, m/z	t_R_, Min	Fragment Ions, m/z	Compounds	Class of Compound	Reference
1.	132.94	0.78	87 (100%)	malic acid	hydroxy acid	Vieira et al. [30]
2.	136.55	0.98	91 (100%)	protocatechuic aldehyde	phenolic aldehyde	Sanz et al. [31]
3.	146.94	0.87	87 (100%)	(–)-citramalic acid *	organic acid	
4.	160.92	1.39	143 (100%), 115, 97, 87, 71	1,1,2-ethanetricarboxylic acid	organic acid	Affes et al. [32]
5.	180.91	1.91	137 (100%), 113	dihydrocaffeic acid	phenolic acid	Pinto et al. [33]
6.	187.05	6.76	125 (100%)	azelaic acid	fatty acid	Liu et al. [34]
7.	205.97	1.54	162, 146 (100%)	unknown		
8.	208.98	6.49	165 (100%), 137	hydroxyferulic acid	phenolic acid	Pinto et al. [33]
9.	211.01	8.51	167 (100%), 149	carboxyvanillic acid	phenolic acid	Wang et al. [35]
10.	224.93 [M–H+ HCOOH+ HOH]^−^	0.96	179 (100%), 101	3- or 4-hydroxy-2-oxoglutaric acid *	gamma-keto acid	
11.	225.04 [M–H+ HCOOH]^−^	0.59	179 (100%), 149, 113, 89	caffeic acid *	phenolic acid	
12.	227.04	5.50	183, 95	*cis*-resveratrol	stilbenoid	Nie et al. [36]
13.	265.10	10.12	97 (100%)	oxidized fatty acid *	fatty acid	
14.	271.19	12.51	253, 225 (100%)	pinobanksin	flavanonol	Gardana et al. [37]
15.	279.16	10.68	97 (100%)	linoleic acid *	fatty acid	
16.	287.18	8.46	269 (100%), 221, 211	fustin	flavanonol	Destandau et al. [38]
17.	289.07	7.05	261 (100%), 215, 173	unknown		
18.	293.09	9.30	236 (100%), 221, 193	hydroxy-octadecatrienoic acid	fatty acid	Elgendi et al. [39]
19.	295.21	10.91	277 (100%), 227, 195	hydroxy-octadecadienoic acid	fatty acid	Elgendi et al. [39]
20.	301.06	7.29	273 (100%), 179, 151, 107	quercetin	flavonol	Vuković et al. [40]
21.	313.23	9.88	295 (100%), 277, 233, 195, 183, 127	dihydroxy-octadecenoic acid	fatty acid	Boškov et al. [20]
22.	315.29	10.49	301, 297 (100%), 279, 171	isorhamnetin derivative	flavonol	Ben Said et al. [41]
23.	326.83	5.37	281 (100%), 237, 211, 189, 164	unknown		
24.	327.17	8.04	291 (100%), 281, 240, 230, 209, 171	oxo-dihydroxy-octadecenoic acid	fatty acid	Grati et al. [42]
25.	329.16	8.28	311, 293, 281, 229 (100%), 211, 183, 171, 165, 127	11,12,13-trihydroxyoctadecenoic acid	fatty acid	Fahmy et al. [43]
26.	339.06	8.19	324 (100%), 253	unknown		
27.	341.15	7.69	326 (100%), 305, 254	unknown		
28.	345.23	7.89	327, 309, 283, 267 (100%), 213, 185, 139	unknown		
29.	401.05	7.86	367, 357 (100%), 329	apigenin-pentosyl-hexosyl derivative	flavone	Grati et al. [42]
30.	405.03	7.54	361 (100%), 333, 261	unknown		
31.	427.21	12.68	383, 355 (100%), 175	juglanoside D derivative	quinone	Żurek et al. [44]
32.	429.12	9.49	401 (100%), 383, 357, 313	apigenin-pentosyl-hexosyl derivative	flavone	Grati et al. [42]
33.	431.13	5.47	387, 233, 224, 179 (100%), 149	caffeic acid derivative *	phenolic acid	
34.	457.19	2.66	281, 237 (100%), 175	dihydrophaseic acid derivative	terpenoid	Sheng et al. [45]
35.	469.19	7.21	423 (100%), 291	valenoic acid dilactone	tannin	Sheng et al. [45]
36.	503.05 [M–H+ HCOOH]^−^	6.84	457 (100%)	dihydrophaseic acid derivative	terpenoid	Sheng et al. [45]
37.	517.12 [M–H+ HCOOH]^−^	6.82	481, 471, 307 (100%), 163	*p*-coumaric acid rutinoside	phenolic acid	Elgendi et al. [39]
38.	535.12	9.52	517, 491 (100%), 395, 371	*p*-coumaroyl-6′-secologanoside	phenolic acid	Božunović et al. [46]
39.	549.07	9.65	531, 505, 487, 385, 357 (100%), 329, 275	unknown		
40.	577.12	7.00	515, 475, 433 (100%), 307	apigenin-7-(6″-*p*-coumaroylglucoside)	flavone	Petreska et al. [47]
41.	593.22	6.22	828, 578, 416, 355, 327, 284 (100%), 239	kaempferol-3-*O*-rutinoside	flavonol	Božunović et al. [46]
42.	610.20	11.02	565 (100%), 291	unknown		
43.	637.03 [M–H+ HCOOH]^−^	7.36	615, 591 (100%), 573, 283	acacetin-rhamnoglucoside isomer	flavone	Al-Yousef et al. [48]
44.	648.94	12.02	580 (100%)	unknown		
45.	739.27	6.04	635, 593 (100%), 299, 285	kaempferol-3-*O*-robinoside-7-*O*-rhamnoside (robinin)	flavonol	March et al. [49]
46.	767.29	6.73	749, 483, 283, 268 (100%)	kaempferol-3-*O*-(4-coumaroyl)- (feruloyl)-glucoside (isomer)	flavonol	Matkovits et al. [50]
47.	769.04	7.08	723 (100%), 703, 623, 283	unknown		
48.	795.54	9.22	439 (100%), 408	unknown		
49.	840.65	9.25	564, 363 (100%)	unknown		

* https://massbank.eu/MassBank/ (accessed on 5 December 2024).

## Data Availability

The original contributions presented in this study are included in the article. Further inquiries can be directed to the corresponding author.
